# Translation regulatory long non‐coding RNA 1 negatively regulates cell radiosensitivity via the miR‐22‐3p/SP1 axis in non‐small cell lung cancer

**DOI:** 10.1111/crj.13734

**Published:** 2024-01-29

**Authors:** Ming Zhong, Zheng Fang, Weixi Guo, Xiuyi Yu

**Affiliations:** ^1^ Department of Thoracic Surgery The First Affiliated Hospital of Xiamen University Xiamen China

**Keywords:** DNA damage repair, ionizing radiation, lncRNA TRERNA1, miR‐22‐3p, non‐small cell lung cancer, radiosensitivity, SP1, γ‐H2AX

## Abstract

**Objective:**

Non‐small cell lung cancer (NSCLC) occupies 85% of lung cancer. Long non‐coding RNAs (LncRNAs) can regulate the radiosensitivity of cancers. This study explored the mechanism of lncRNA TRERNA1 in the radiosensitivity of NSCLC cells.

**Methods:**

LncRNA TRERNA1 level in NSCLC cell lines was determined. NSCLC cell radiation tolerance was measured. TRERNA1 expression was silenced or overexpressed in A549/HCC827 cells with the highest/lowest radiation tolerance, respectively. The contents of γ‐H2AX and SA‐β‐gal in NSCLC cells after radiation induction were detected. The targeted binding of TRERNA1 to miR‐22‐3p and miR‐22‐3p to SP1 were verified by dual‐luciferase assay. SP1 expression were detected. Functional rescue experiments were implemented to confirm the roles of miR‐22‐3p and SP1 in the regulatory mechanism of TRERNA1.

**Results:**

TRERNA1 was upregulated in NSCLC cells. TRERNA1 silencing enhanced radiosensitivity of NSCLC cells. TRERNA1 silencing elevated the contents of γ‐H2AX and SA‐β‐gal in A549 cells after radiation induction, while TRERNA1 overexpression showed an opposite trend in HCC827 cells. There were targeting relationships between TRERNA1 and miR‐22‐3p, and miR‐22‐3p and SP1. miR‐22‐3p repression or SP1 overexpression abolished the effects of TRERNA1 silencing.

**Conclusion:**

TRERNA1 silencing enhanced radiosensitivity of NSCLC cells via the miR‐22‐3p/SP1 axis. This study may offer new targets for NSCLC treatment.

## Graphical abstract

Non‐small cell lung cancer (NSCLC) accounts for 85% of lung cancer. Long non‐coding RNAs (LncRNAs) can regulate the radiosensitivity of cancers. This study explored the mechanism of lncRNA TRERNA1 in the radiosensitivity of NSCLC cells.

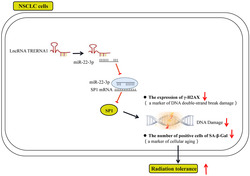

## 1 INTRODUCTION

As one of the most prevalent malignancies, lung cancer has a high mortality.^1^ Among which, non‐small cell lung cancer (NSCLC) is the most prevalent histologic type of lung cancer, making up 85%–90% of lung cancers, with large cell carcinoma, adenocarcinoma, and squamous cell carcinoma being the three leading subtypes.^2^ Radiation therapy is a momentous remedy for NSCLC.^3^ However, the low radiosensitivity of lung cancer cells and the fact that the radiation dose targeted to the tumor cannot be increased indefinitely without damaging the surrounding normal tissues are the main factors affecting the effectiveness of radiation therapy.^4^ Due to these limitations, survival rates for NSCLC patients after treatment in recent decades have remained suboptimal and have not significantly ameliorated.^5^ Therefore, it is of vital importance to enhance radiation therapy sensitivity for NSCLC, identify the molecular mechanisms of NSCLC radiation therapy resistance, and provide sensitization targets for RT for the clinical treatment of NSCLC.

DNA damage response is a pivotal anti‐cancer barrier that can improve tumor sensitivity to RT by regulating DNA damage response.^6^ DNA double‐strand break (DSB) participates in DNA damage repair and is an important factor in cellular radiosensitivity.^7^ It was reported that radiosensitivity of NSCLC cells could be enhanced by delaying DSB repair.^4^ This study intended to probe an effective approach for the treatment of NSCLC from the perspective of DSB and radiation therapy.

Long non‐coding RNAs (LncRNAs) belongs to noncoding RNA transcripts with over 200 nucleotides.^8^ LncRNAs regulate radiosensitivity via substantial mechanisms, for instance, DNA damage repair, apoptosis, cell‐cycle arrest, epithelial mesenchymal transition, and autophagy.^9^ LncRNA translation regulatory long non‐coding RNA 1 (TRERNA1) is deemed to be a carcinogenic factor in many cancers and is high in the NSCLC patient tumor tissues.^10^ Elevated TRERNA1 associated with HIF‐1α activation has something to do with poor prognosis in hepatocellular carcinoma.^11^ Nevertheless, whether TRERNA1 affects NSCLC radiosensitivity by regulating DNA damage has not been reported.

Mechanically, lncRNA transcripts bind to microRNA (miRNA) response elements competitively to mutually manipulate gene expression, thus serving as competing endogenous RNAs (ceRNAs).^12^, ^13^ LncRNA ANRIL pitched in the manipulation of tumor radiosensitivity through the ceRNA mechanism.^14^, ^15^ The lncRNA DLX6‐AS1/miR‐16/BMI1 axis manipulates DNA damage and activates apoptosis and autophagy of NSCLC cells.^16^ It has been documented that miR‐22‐3p expression was poor in NSCLC and act in the modulation of proliferation, invasion, and migration of NSCLC cells.^17^, ^18^ Increased radiosensitivity could be achieved by miR‐22 targeting WRNIP1 in small cell lung cancer.^19^ Moreover, TRERNA1 has been reported to modulate NRAS via sponging miR‐22‐3p.^20^ Whether miR‐22‐3p could affect NSCLC cell radiosensitivity by regulating DNA damage and whether TRERNA1 could affect NSCLC cell radiosensitivity by sponging miR‐22‐3p to regulate DNA damage have not been investigated.

Previous studies showed that specific protein 1 (SP1) comes into play in the progression of tumor, modulates radiosensitivity and tumor suppressor protein p53‐binding protein 1, and acts in DSB repair and the regulation of tumor radiosensitivity.^21^, ^22^, ^23^ What's more, a prior study depicted that SP1 makes a difference in DNA repair and radiosensitivity of NSCLC.^24^ Based on the above references, we hypothesize that TRERNA1 may affect radiation‐induced DSB repair by sponging miR‐22‐3p to manipulate SP1 expression, thereby affecting NSCLC cell radiosensitivity. This study shall confer novel insights for the radiotherapy for NSCLC patients.

## 2 MATERIAL AND METHODS

### 2.1 Cell culture

Human normal lung cell line HBE and NSCLC cell lines (A549, H1299, H292, H3122, HCC827, and PC9; Shanghai Institute of Cell Biology, Shanghai, China) were fostered in Dulbecco's modified Eagle's medium containing 10% fetal bovine serum, 100 μg/mL streptomycin, and 100 U/mL penicillin, with 5% CO_2_ at 37°C.

### 2.2 Cell treatment and grouping

HCC827 cells were arranged to three groups: blank group, oe‐NC group (transfected with PCDNA 3.1 empty vector), and oe‐TRERNA1 group (transfected with pcDNA 3.1‐TRERNA1 plasmid). A549 cells were assigned to seven groups: blank group, si‐TRERNA1 group (transfected with TRERNA1 siRNA), si‐NC group (transfected with siRNA NC), si‐TRERNA1 + miR‐inhi group (simultaneously transfected with TRERNA1 siRNA and miR‐22‐3p inhibitor), si‐TRERNA1 + miR‐NC group (simultaneously transfected with TRERNA1 siRNA and inhibitor NC), si‐TRERNA1 + oe‐SP1 group (simultaneously transfected with TRERNA1 siRNA and pcDNA 3.1‐SP1 plasmid), and si‐TRERNA1 + oe‐NC group (simultaneously transfected with TRERNA1 siRNA and pcDNA 3.1 empty vector).

All the transfectants were provided by Shanghai GenePharma Co., Ltd. (Shanghai, China). The transfection was operated strictly abided by the manuals of Lipofectamine™ 2000 (Invitrogen). The final concentration of each transfectants after transfection was 50 nmol/L. After 48 h, the follow‐up experiments were performed.

### 2.3 Reverse transcription quantitative polymerase chain reaction (RT‐qPCR)

TRIzol (Invitrogen) was adopted to extract the total RNA, and the purity and concentration of RNA were evaluated using an ultramicro spectrophotometer (Spectral Instrument, Shanghai, China). Then, RNA (1 μg) was reversely transcribed into cDNA using a reverse transcription kit (Thermo Fisher Scientific Inc., Waltham, MA, USA). The ABI PRISM 7900 sequence detection system of SYBR Green II (Takara Bio Inc., Kyoto, Japan) was used for quantitative PCR. 2^‐ΔΔCt^ method was applied for gene relative expression. GAPDH and U6 served as the internal reference. All primers were synthesized by Sangon Biotech (Shanghai, China; Table 1).

**TABLE 1:** Primer sequence for RT‐qPCR.

Gene	Forward 5′‐3′	Reverse 5′‐3′
TRERNA1	CCGTTGGCTCCACAAACCT	CAGTGACAGTAGCAGGCATCCT
miR‐22‐3p	AAGCTGCCAGTTGAAGAACTGTA	GCTGTCAACGATACGCTACGTAAC
GAPDH	GCACCGTCAAGGCTGAGAAC	TGGTGAAGACGCCAGTGGA
U6	CGCTTCGGCAGCACATATACTA	CGCTTCACGAATTTGCGTGTCA

### 2.4 Colony formation assay

The 6‐well plates were seeded with NSCLC cells in exponential stage. The cell density in the 0 Gy group, 2 Gy group, 6 Gy group, and 8 Gy group was 0.5 × 10^3^ cells/well, 1.0 × 10^3^ cells/well, 1.5 × 10^3^ cells/well, and 5 × 10^3^ cells/well, respectively. Then, according to the experimental requirements, the NSCLC cells in each corresponding group were irradiated with 0, 2, 6, and 8 Gy for 24 h, and then cultured in the cell incubator for 10–14 days. The cultivation was terminated when there were visible colonies in the plates. Thereafter, cells were fixed with 1 mL paraformaldehyde (4%) for 15 min. Next, cells were stained with 1 mL crystal violet staining solution (0.5%) for 10–30 min. The microscope was implemented to observe 6‐well plates (Olympus Optical Co., Ltd, Tokyo, Japan) to count the number of cell colony which had over 50 cells. Survival fraction = (number of colonies/number of cells plated)_irradiated_/(number of colonies/number of cells plated)_non‐irradiated_.

### 2.5 Immunofluorescence staining

A549/HCC827 cells exposed to 0Gy/8Gy irradiation for 24 h on 4‐chamber culture slides (BD Falcon, Bedford, MA, USA) were fixed, permeabilized, and stained. Then, cells were stained with mouse anti‐γ‐H2AX (ab26350, Abcam Inc., Cambridge, CA, USA) and Alexa Fluor 568‐conjugated anti‐mouse immunoglobulin G (IgG) (1:500, A11004, Invitrogen). Nuclei were counterstained with Hoechst‐33342. About 200 nuclei images were acquired using a Zeiss Axio Observer Z1 invert microscope equipped with an Apo 60X/1.4 oil DICIII objective. Afterwards, images were acquired in AxioVision software (4.7.1.0; Carl Zeiss Microimaging Inc., GmbH, Jena; Germany). Finally, the numbers of γ‐H2AX for each cell were accounted, averaged, and presented as γ‐H2AX cell.

### 2.6 Senescence‐associated β‐galactosidase (SA‐β‐gal) staining

Cells exposed to dimethyl sulphoxide solution for 2 h were subjected to 0 or 8 Gy radiation. A SA‐β‐gal staining kit (Cell Signaling Technology, Beverly, MA, USA) was adopted for SA‐β‐gal activity 6 days after irradiation. After that, cell senescence (blue‐stained) was identified with a light microscope. SA‐β‐gal‐positive cell percentage was quantified from six different fields.

### 2.7 Dual‐luciferase report assay

The wild‐type (pmirGLO‐TERRNA1‐WT/SP1‐WT) and mutant‐type (pmirGLO‐TERRNA1‐MUT/SP1 MUT) 3’‐UTR of TRERNA1/SP1 were independently cloned into the pmirGLO luciferase report vector comprising renilla luciferase gene (Promega, Madison, WI, USA). In luciferase detection, A549 cells were cotransfected with miR‐22‐3p mimic or mimic NC and luciferase report plasmids using Lipofectamine 2000. After 48‐h transfection, following the provided agreement, the dual‐luciferase report detection system (Promega) was conducted to measure firefly luciferase activity. The firefly luciferase of each well was normalized to renilla luciferase activity.

### 2.8 Western blot analysis

The cells were added into radio‐immunoprecipitation assay cell lysis buffer (KeyGEN Biotech, Nanjing, Jiangsu, China) and homogenized. Subsequently, cell were centrifuged to extract the protein. Protein concentration was assessed by bicinchoninic acid method, and 30 μg protein samples were adopted for sodium dodecyl sulfate polyacrylamide gel electrophoresis. Later, the proteins were transferred onto the membranes, sealed with skim milk (5%) powder for 2 h at room temperature, and cultivated with the primary antibody SP1 (1:1000, ab231778, Abcam) at 4°C overnight. Afterwards, the membranes were washed, fostered with diluted secondary antibody IgG (1:2000, ab205718, Abcam) for 1 h. After the membranes were washed again, the membranes were immersed in the exposure solution and dispersed evenly, and then put onto the machine for exposure. The gray value of exposure results was statistically analyzed, with GAPDH (1:10000, ab181602, Abcam) as an internal reference.

### 2.9 Statistical analysis

Statistical analysis was implemented using SPSS21.0 (IBM Corp. Armonk, NY, USA) and GraphPad Prism 8.01 (GraphPad Software, San Diego, CA, USA). The measurement data were exhibited as mean ± standard deviation. The *t*‐test was applied for data analysis between two groups. The one‐way analysis of variance (ANOVA) was applied to analyze the comparisons among multi‐groups, followed by Tukey's multiple comparisons test. *p*‐Value was acquired by two‐tailed tests and *p* < 0.05 meant a statistical difference.

## 3 RESULTS

### 3.1 TRERNA1 was highly expressed in NSCLC cells and enhanced radiation tolerance

**FIGURE 1:**
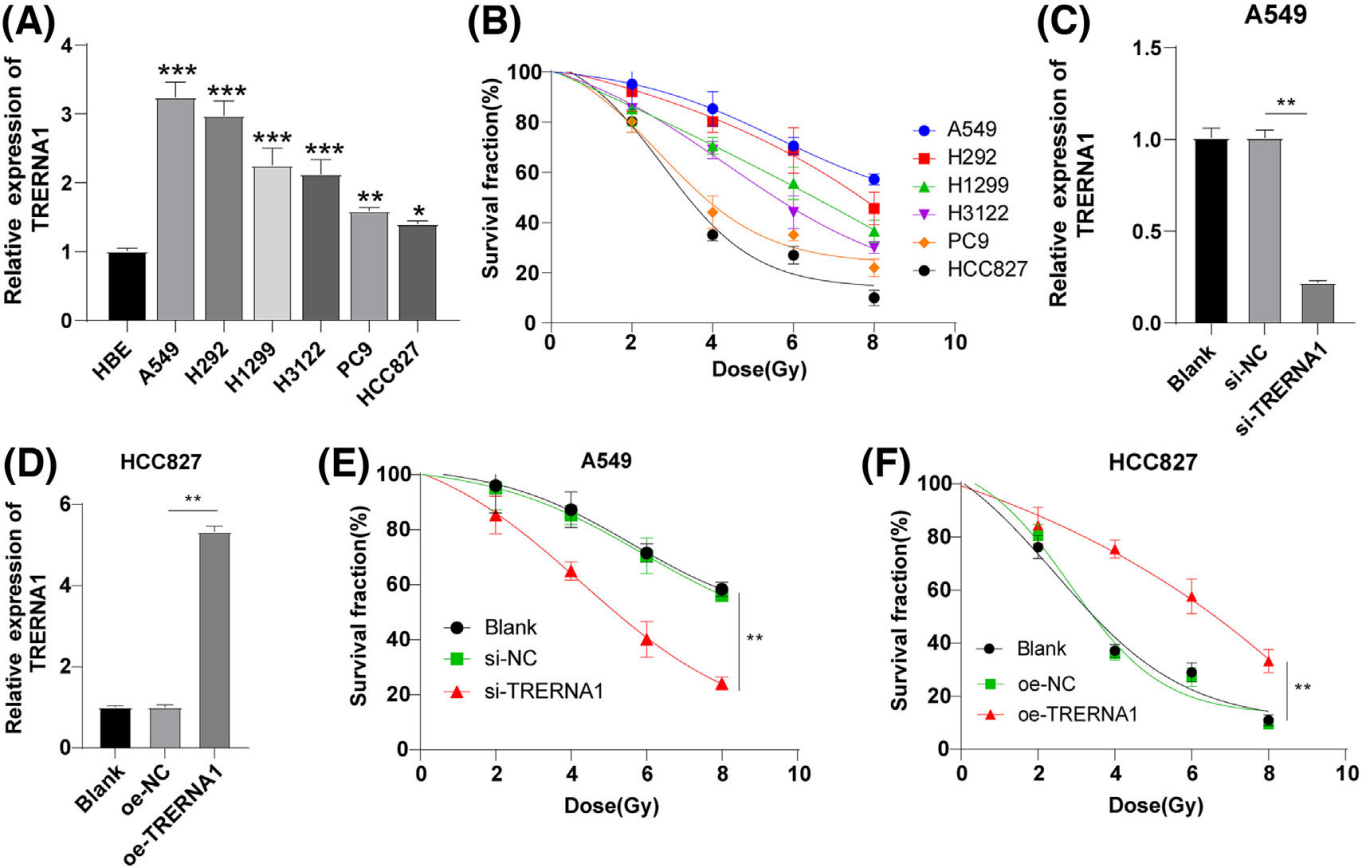
TRERNA1 was highly expressed in NSCLC cells and enhanced radiation tolerance. (A) TRERNA1 expression was detected using RT‐qPCR. (B) The radiation tolerance of NSCLC cells was detected using colony formation assay. TRERNA1 was inhibited in A549 cells and overexpressed in HCC827 cells. (C, D) TRERNA1 expression was detected using RT‐qPCR. (E, F) The radiation tolerance of NSCLC cells was detected using colony formation assay. TRERNA1 (translation regulatory long non‐coding RNA 1), si (siRNA), NC (negative control), oe (overexpression). The cell experiment was repeated three times independently. Data were presented as mean ± standard deviation and analyzed using one‐way ANOVA, followed by Tukey's multiple comparisons test, *p < 0.05; **p < 0. 01; ***p < 0.001.

RT‐qPCR results displayed that lncRNA TRERNA1 was generally upregulated in NSCLC cells (all *p* < 0.05), with its expression A549 > H292 > H1299 > H3122 > PC9 > HCC827 in NSCLC cell lines (Figure 1A). Subsequently, we detected the radiation tolerance of these six NSCLC cell lines using colony formation assay. The results showed that the survival rate of the cell lines with lncRNA TRERNA1 high expression (A549/H292) was higher than that of cell lines with lncRNA TRERNA1 low expression (PC9/HCC827; Figure 1B), indicating that TRERNA1 was positively correlated with radiation tolerance of NSCLC cells. To further probe the role of TRERNA1 on radiation tolerance of NSCLC cells, we transfected TRERNA1 siRNA into A549 cells with relative high TRERNA1 expression (si‐TRERNA1 group) to silence TRERNA1 expression and transfected TRERNA1 into HCC827 cells with relative low TRERNA1 expression (oe‐TRERNA1 group) to overexpress TRERNA1. RT‐qPCR manifested that, relative to the si‐NC group, TRERNA1 expression was significantly diminished in the si‐TRERNA1 group and elevated in the oe‐TRERNA1 group relative to the oe‐NC group (all *p* < 0.01) (Figure 1C,D), confirming successful cell transfection. The results of colony formation assay unraveled that inhibition of TRERNA1 prominently enhanced the radiosensitivity of A549 cells (*p* < 0.01; Figure 1E), while TRERNA1 overexpression reduced the radiosensitivity of HCC827 cells (*p* < 0.01; Figure 1F). The aforesaid results depicted that TRERNA1 was highly expressed in NSCLC cells and enhanced radiation tolerance.

### 3.2 TRERNA1 silencing promoted radiation‐induced DSB and cell aging in NSCLC cells

**FIGURE 2:**
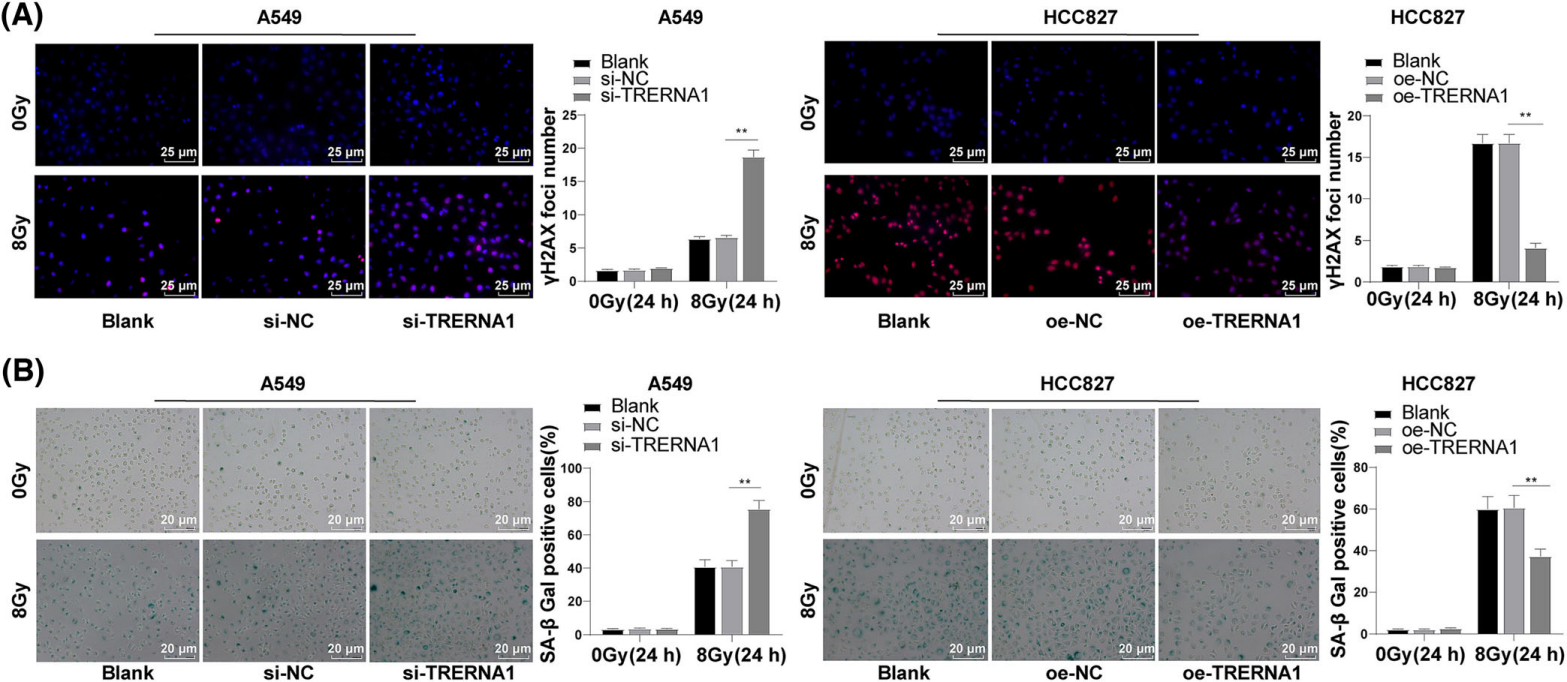
TRERNA1 silencing promoted radiation‐induced double‐strand break (DSB) and cell injury in NSCLC cells. TRERNA1 expression was inhibited in A549 cells and overexpressed in HCC827 cells. (A) The content of DSB marker γ‐H2AX was detected using immunofluorescence after 24 h of 0/8 Gy radiation induction. (B) The number of SA‐β‐gal‐positive cells was detected using SA‐β‐gal assay after 24 h of 0/8 Gy IR. TRERNA1 (translation regulatory long non‐coding RNA 1), si (siRNA), NC (negative control), oe (overexpression). The cell experiment was repeated three times independently. Data were presented as mean ± standard deviation and analyzed using one‐way ANOVA, followed by Tukey's multiple comparisons test, **p < 0.01.

The expression of post‐radiation damage marker γ‐H2AX in NSCLC cells was evaluated by immunofluorescence staining after silencing or overexpression of TRERNA1. TRERNA1 silencing led to a distinct increase in γ‐H2AX expression in A549 cells after 24 h of radiation induction, and overexpression of TRERNA1 sparked off a dramatic decrease in the expression of γ‐H2AX in HCC827 cells after 24 h of radiation induction (all *p* < 0.01; Figure 2A). Also, we evaluated the impact of TRERNA1 on radiation‐treated cell DNA injury by measuring the number of SA‐β‐Gal‐positive cells. TRERNA1 silencing observably boosted the number of SA‐β‐Gal‐positive cells in A549 cells, while overexpression of TRERNA1 showed an opposite trend in HCC827 cells (all *p* < 0.01; Figure 2B). Collectively, TRERNA1 silencing enhanced radiation‐induced NSCLC cell DSB and injury, while TRERNA1 overexpression repressed radiation‐induced NSCLC cell DSB and injury.

### 3.3 TRERNA1 regulated the radiosensitivity of NSCLC cells by suppressing miR‐22‐3p expression

**FIGURE 3:**
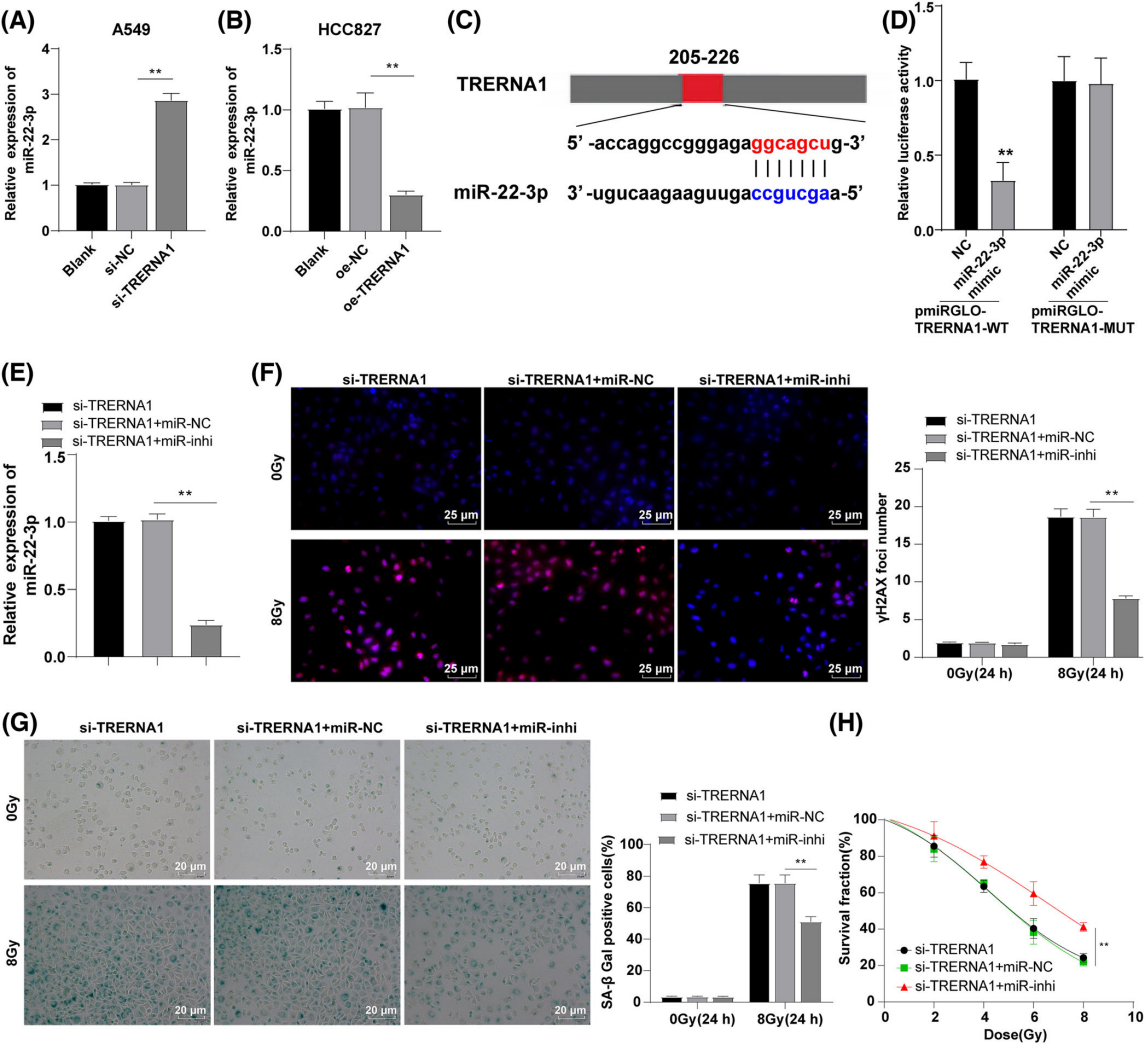
Silencing of TRERNA1 promoted radiation‐induced DSB and cell aging and enhanced radiosensitivity of NSCLC cells by repressing miR‐22‐3p expression. (A‐B) miR‐22‐3p expression was detected using RT‐qPCR. (C) The binding relationship between TRERNA1 and miR‐22‐3p was verified using the dual‐luciferase assay. TRERNA1 was knocked down and miR‐22‐3p was inhibited in A549 cells, and (E) miR‐22‐3p expression was detected using RT‐qPCR. (F) The content of γ‐H2AX was detected using immunofluorescence after 24 h of 0/8 Gy IR. (G) The number of SA‐β‐gal‐positive cells was detected using SA‐β‐gal assay after 24 h of 0/8 Gy IR. (H) The radiation tolerance of NSCLC cells was detected using colony formation assay. TRERNA1 (translation regulatory long non‐coding RNA 1), si (siRNA), NC (negative control), oe (overexpression), miR (microRNA), inhi (inhibitor). The cell experiment was repeated three times independently. Data were presented as mean ± standard deviation and analyzed using *T* test between two groups in panel (D), and using one‐way ANOVA among groups, followed by Tukey's multiple comparisons test, **p < 0.01.

miR‐22‐3p expression in A549 and HCC827 cells were detected. TRERNA1 silencing significantly increased the miR‐22‐3p expression in A549 cells (*p* < 0.01; Figure 3A), while overexpression of TRERNA1 decreased miR‐22‐3p expression in HCC827 cells (*p* < 0.01; Figure 3B). Subsequently, A549 cells were selected for further experiments. Based on the previously reported targeted binding sites between TRERNA1 and miR‐22‐3p^20^ (Figure 3C), the binding relationship between TRERNA1 and miR‐22‐3p was verified using the dual‐luciferase assay (*p* < 0.01; Figure 3D). Briefly, lncRNA TRERNA1 inhibited miR‐22‐3p expression. Furthermore, we silenced TRERNA1 and repressed miR‐22‐3p expression together in A549 cells for joint experiment (si‐TRERNA1 + miR‐inhi group). RT‐qPCR showed that the si‐TRERNA1 + miR‐inhi group had significantly reduced miR‐22‐3p expression versus the si‐TRERNA1 + miR‐NC group (*p* < 0.01; Figure 3E). Subsequently, we detected the expression levels of DSB biomarker γ‐H2AX and cell aging biomarker SA‐β‐Gal after radiation damage in cells of each treatment group. Inhibition of miR‐22‐3p partially abolished the regulatory role of TRERNA1 silencing on γ‐H2AX and SA‐β‐Gal (all *p* < 0.01; Figure 3F,G), and also partially reversed the facilitating effect of TRERNA1 silencing on radiosensitivity (*p* < 0.01; Figure 3H). These results demonstrated that TRERNA1 silencing promoted radiation‐induced DSB and cell aging via curbing miR‐22‐3p expression, thus enhancing radiosensitivity of NSCLC cells.

### 3.4 miR‐22‐3p targeted SP1 expression

**FIGURE 4:**
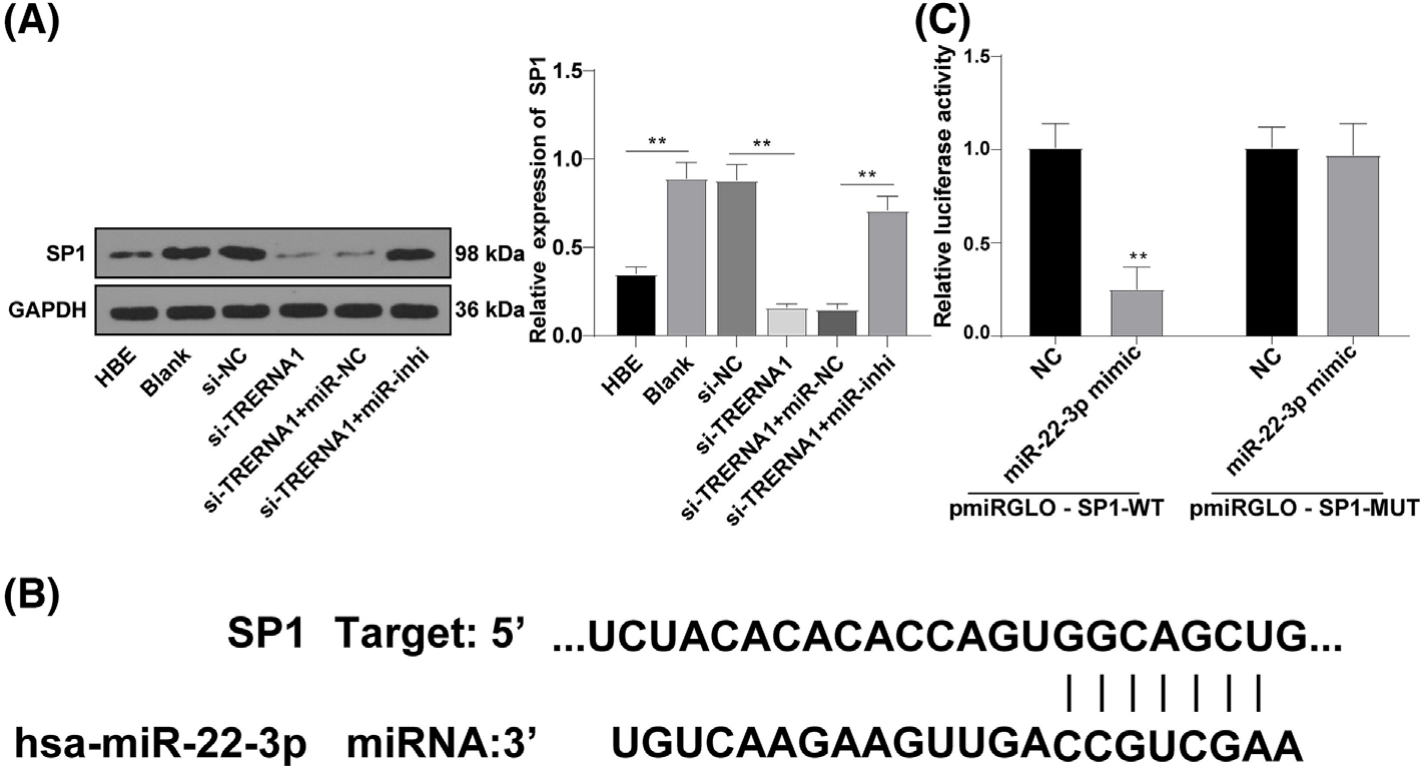
miR‐22‐3p targeted SP1. (A) SP1 expression was detected using Western blot. (B) The targeted binding sites between miR‐22‐3p and SP1 were predicted using TargetScan database. (C) Binding relationship between miR‐22‐3p and SP1 was verified using the dual‐luciferase assay. The cell experiment was repeated three times independently.SP1 (specific protein 1), TRERNA1 (translation regulatory long non‐coding RNA 1), si (siRNA), NC (negative control), miR (microRNA), inhi (inhibitor) Data were presented as mean ± standard deviation and analyzed using *t*‐test between two groups in panel (C), and using one‐way ANOVA among groups, followed by Tukey's multiple comparisons test, **p < 0.01.

To further investigate whether SP1 could participate in the manipulation of radiosensitivity in NSCLC cells in the possible regulatory pathway of TRERNA1 and miR‐22‐3p, SP1 expression in A549 cells was determined by western blot. The results found that TRERNA1 silencing significantly reduced SP1 expression, while further miR‐22‐3p repression increased SP1 expression (all *p* < 0.01; Figure 4A). TargetScan database predicted that miR‐22‐3p had targeted binding sites with SP1 (Figure 4B). Meanwhile, the binding relationship between miR‐22‐3p and SP1 was verified using dual‐luciferase assay (*p* < 0.01; Figure 4C). Together, miR‐22‐3p targeted SP1 in A549 cells.

### 3.5 Overexpression of SP1 partially reversed the effect of TRERNA1 silencing on enhancing radiosensitivity of NSCLC cells

**FIGURE 5:**
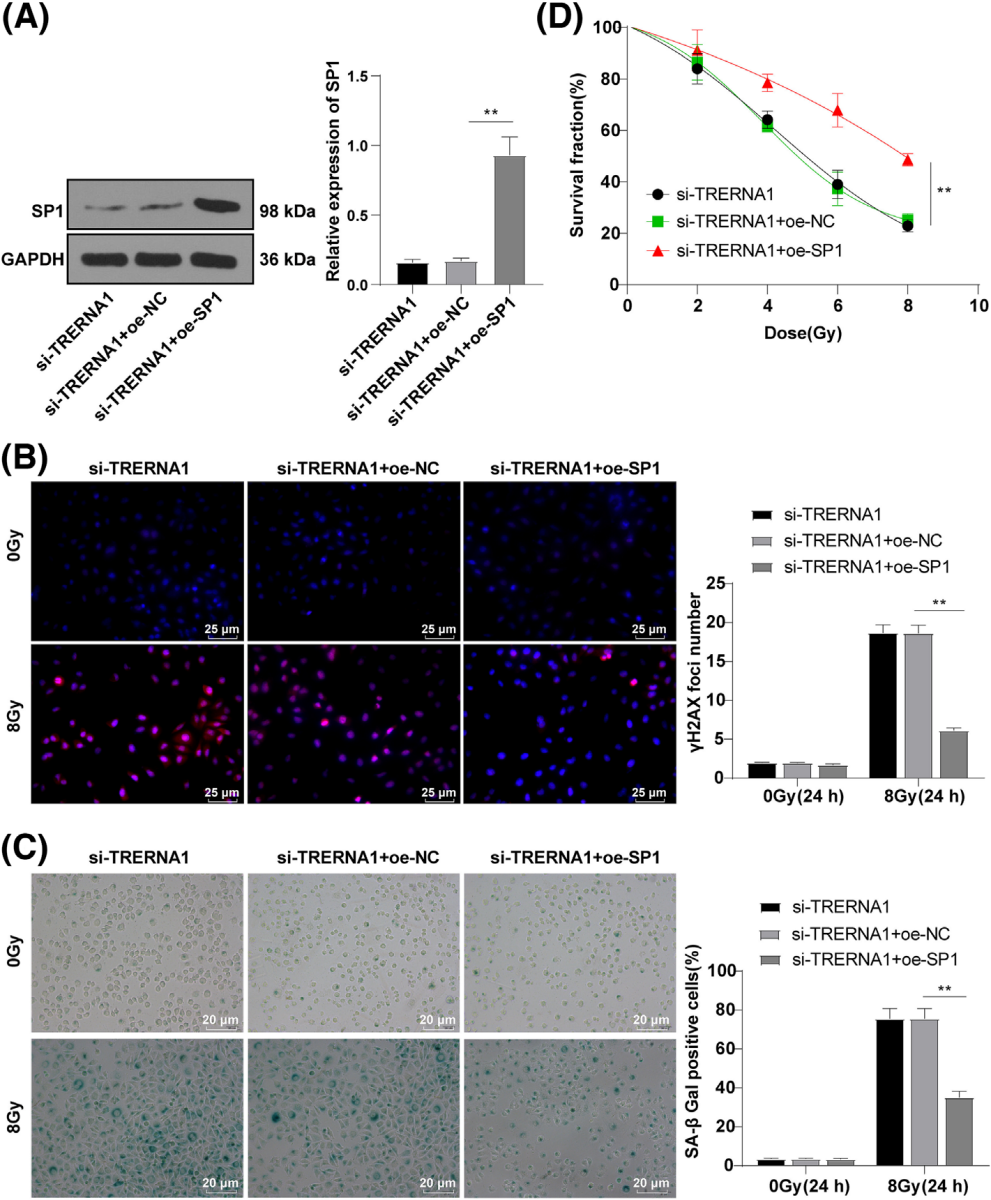
Overexpression of SP1 reversed the enhancing effect of TRERNA1 knockdown on radiosensitivity of NSCLC cells. TRERNA1 was silenced and SP1 was overexpressed in A549 cells. (A) SP1 expression was detected using Western blot. (B) The content of γ‐H2AX was detected using immunofluorescence after 24 h of 0/8 Gy IR. (C) The number of SA‐β‐gal‐positive cells was detected using SA‐β‐gal assay after 24 h of 0/8 Gy IR. (D) The radiation tolerance of NSCLC cells was detected using colony formation assay. SP1(specific protein 1), TRERNA1 (translation regulatory long non‐coding RNA 1), si (siRNA), NC (negative control), oe (overexpression) The cell experiment was repeated three times independently. Data were presented as mean ± standard deviation and analyzed using one‐way ANOVA, followed by Tukey's multiple comparisons test, **p < 0.01.

Finally, we silenced TRERNA1 and overexpressed SP1 together in A549 cells (si‐TRERNA1 + oe‐SP1 group). Compared with the si‐TRERNA1 + oe‐NC group, SP1 expression in the si‐TRERNA1 + oe‐SP1 group was dramatically increased (*p* < 0.01; Figure 5A). Subsequently, we detected the expressions of γ‐H2AX and SA‐β‐Gal, and the radiosensitivity of cells in each group. The results showed that SP1 overexpression reversed the effect of TRERNA1 knockout on enhancing radiosensitivity of NSCLC cells (all *p* < 0.01; Figure 5B–D).

## 4 DISCUSSION

Radiotherapy is commonly used in curative and palliative intention in numerous clinical NSCLC cases.^25^ Nonetheless, radiotherapy efficacy is largely subjected to radioresistance, ultimately translating into relapse and metastasis.^26^ LncRNA TRERNA1 can regulate the development of NSCLC by targeting Forkhead box L1.^10^ This study demonstrated that lncRNA TRERNA1 negatively manipulated the radiosensitivity of NSCLC cells via the miR‐22‐3p/SP1 axis.

LncRNA TRERNA1 is accepted as a fatal oncogene implicated in a variety of malignancies.^27^ TRERNA1 expression is hoisted in NSCLC cells/tissues, and upregulated TRERNA1 is concerned with poor prognosis.^10^ Our data suggested that TRERNA1 was highly expressed in NSCLC cells, and TRERNA1 was favorably interrelated with radiation tolerance of NSCLC cells. To further probe the role of LncRNA TRERNA1 on radiation tolerance of NSCLC cells, we transfected TRERNA1 siRNA into A549 cells and TRERNA1 into HCC827 cells. TRERNA1 silencing remarkably boosted the radiosensitivity of A549 cells, while TRERNA1 expression reduced the radiosensitivity of HCC827 cells. Sun et al. have also indicated that TRERNA1 expression was upregulated in ependymomas cells.^15^ Wu et al. have elucidated that downregulation of TRERNA1 represses the migrating, invasive, and tumorigenic abilities in gastric cancer cells.^28^

The DSB induced by radiation may be the potential mechanism of some anti‐cancer treatments.^29^ Nevertheless, tumor cells can still recover the led DSB, thus crippling the therapeutic effect; therefore, inhibition of DSB repair becomes one of suggested mechanisms to sensitize tumor cells to radiation therapy.^30^ γ‐H2AX is a novel biomarker for DNA DSB, and the detection of γ‐H2AX allows the evaluation of DNA damage.^31^ SA‐β‐Gal activity is commonly utilized to measure the cellular senescence.^32^ Our results exhibited that TRERNA1 silencing increased the expression of γ‐H2AX, the number of SA‐β‐Gal‐positive cells, and the comet tail length in A549 cells after 24 h of 6 Gy radiation, while overexpression of TRERNA1 showed an opposite trend in HCC827 cells. It was suggested that TRERNA1 silencing inhibited the repair of radiation‐led DSB in NSCLC cells.

A prior study revealed the presence of target binding sites for TRERNA1 and miR‐22‐3p.^20^ Downregulated miR‐22‐3p expression can be detected in NSCLC cells/tissues, and miR‐22‐3p hampers NSCLC progression.^17^ miR‐22‐3p overexpression is documented to reduce the migrating property of small‐cell lung cancer cells, and miR‐22‐3p could boost its radiosensitivity by targeting WRNIP1.^19^ In this study, our results revealed that TRERNA1 targeted miR‐22‐3p expression. Inhibition of miR‐22‐3p diminished the regulatory role of TRERNA1 on γ‐H2AX and SA‐β‐Gal, and also partially reversed the facilitating role of TRERNA1 silencing on radiosensitivity. miR‐22‐3p and MDC1 form a loop to facilitate DNA repair, thus curbing DNA damage.^33^ Briefly, lncRNA TRERNA1 regulated the DSB repair after radiation induction and then affected the radiosensitivity of NSCLC cells by sponging miR‐22‐3p. Subsequently, we shifted to determining the target genes regulated by lncRNA TRERNA1/miR‐22‐3p. The target genes of miR‐22‐3p were predicted, and the co‐expression of genes was searched. SP1 is a critical component in the detection of and response to radiation‐induced DSB.^21^ Inhibition of SP1 expression curtails DNA repair and enhances the radiosensitivity of NSCLC.^24^ Hence, we detected SP1 expression in A549 cells of each treatment group, and found that TRERNA1 silencing significantly reduced SP1 expression, while inhibition of miR‐22‐3p elevated SP1 expression. SP1 overexpression abolished the impact of TRERNA1 on radiosensitivity of NSCLC cells. Downregulation of SP1 increased sensitivity to ionizing radiation.^22^ Moreover, A549 cells of different groups were selected to establish the murine model of NSCLC. si‐TRERNA1‐treated mice showed reduced TRERNA1 and SP1 expressions and enhanced miR‐22‐3p expression. The model mice were subjected to radiation induction. The in vivo experiments confirmed that TRERNA1 silencing significantly enhanced the inhibitory role of radiation on tumor growth in nude mice.

To sum up, lncRNA TRERNA1 regulated radiation‐induced DSB and then affected radiosensitivity of NSCLC cells via the miR‐22‐3p/SP1 axis. However, this study lacked of clinical data, and did not select more cell lines to perform more comprehensive cell experiments. Whether other miRNAs or target genes participated in the mechanism of lncRNA TRERNA1 regulating radiosensitivity of NSCLC cells remained unknown. In the future, we shall conduct clinical research and select more cell lines to perform comprehensive cell experiments. Also, we will focus on the other potential miRNAs, target genes, and signaling pathways manipulated by the lncRNA TRERNA1/miR‐22‐3p/SP1 axis in radiosensitivity of NSCLC cells.

## AUTHOR CONTRIBUTIONS

Ming Zhong contributed to the study concepts and study design. Zheng Fang contributed to the literature research. Ming Zhong, Zheng Fang, and Weixi Guo contributed to the experimental studies and data acquisition. Ming Zhong and Xiuyi Yu contributed to the data analysis and statistical analysis. Ming Zhong contributed to the manuscript preparation and Xiuyi Yu contributed to the manuscript editing and review. All authors read and approved the final manuscript.

## CONFLICT OF INTEREST STATEMENT

The authors have no conflicts of interest to declare.

## ETHICS APPROVAL

Not applicable.

## Data Availability

All data generated or analyzed during this study are included in this article. Further enquiries can be directed to the corresponding author.
